# Thoracoscopy for Pediatric Thoracic Neurogenic Tumors—A European Multi-Center Study

**DOI:** 10.3390/cancers15225467

**Published:** 2023-11-18

**Authors:** Jean François Lecompte, Sabine Sarnacki, Sabine Irtan, Christian Piolat, Aurélien Scalabre, Isabelle Talon, Julien Rod, Nicoleta Panait, Gregory Rodesch, Ana Lourdes Luis Huertas, Olivier Abbo, Martine Demarche, Edouard Habonimana, Quentin Ballouhey, Dominique Valteau-Couanet, Florent Guérin

**Affiliations:** 1 Fondation Lenval, University Nice Côte d’Azur, 06200 Nice, France; 2 Necker Enfants Malade Hospital, Université Paris-Cité, GHU Centre Assistance Publique-Hôpitaux de Paris (AP-HP), 75015 Paris, France; 3 Trousseau Hospital, Université Paris-Sorbonne, GHU-Paris Sorbonne Assistance Publique-Hôpitaux de Paris (AP-HP), 75012 Paris, France; sabine.irtan@aphp.fr; 4 Grenoble-Alpes University Hospital, 38700 La Tronche, France; cpiolat@chu-grenoble.fr; 5 Saint-Etienne University Hospital, 42270 Saint-Priest-en-Jarez, France; aurelien.scalabre@chu-st-etienne.fr; 6 Hopital Hautepierre, CHRU Strasbourg, 67200 Strasbourg, France; 7 CHU Côte de Nâcre, 14033 Caen, France; rod-j@chu-caen.fr; 8 La Timone Hospital, Aix-Marseille University, Assistance Publique-Hôpitaux de Marseille (AP-HM), 13005 Marseille, France; 9 Hôpital Universitaire des Enfants Reine Fabiola, 1020 Bruxelles, Belgium; 10 Hospital Universitario Infantil Niño Jesús, 28009 Madrid, Spain; analluiscp@gmail.com; 11 Hôpital des Enfants, CHU de Toulouse, 31300 Toulouse, France; abbo.o@chu-toulouse.fr; 12 Centre Hospitalier Régional Citadelle, 4000 Liege, Belgium; 13 Rennes University Hospital, 35033 Rennes, France; edouard.habonimana@chu-rennes.fr; 14 Hôpital de la mère et de l’enfant, Centre Hospitalier Universitaire de Limoges, 87000 Limoges, France; 15 Gustave Roussy, Paris-Saclay University, 94805 Villejuif, France; 16 Bicêtre Hospital, Paris-Saclay University, GHU Paris Saclay Assistance Publique Hôpitaux de Paris (AP-HP), 94270 Le Kremlin Bicêtre, France

**Keywords:** thoracoscopy, neuroblastoma, tumor, pediatrics

## Abstract

**Simple Summary:**

Surgery remains the main treatment for thoracic neurogenic tumors. However, with the development of the minimally invasive approach, few studies so far have defined the indications for thoracoscopy in these tumors. We conducted a large retrospective multicenter study to define the role of thoracoscopy for neurogenic tumors. We identified 119 patients from 15 centers over 20 years. The median operative time was 2 h, and the length of stay was 4 days. The conversion rate was 11%, the long-term complication rate (Horner’s and back issues) was 11%, the residue rate was 11.8%, the relapse rate was 7.5%, and 4% of patients died of their disease. The risk for conversion was associated with the need for chemotherapy. Complications were related to the IDRFs, the residue rates associated with persisting bronchial or vascular IDRF, and size but not on multivariate analysis. Recurrences were associated with neuroblastoma histology and unfavorable tumor biology.

**Abstract:**

Objectives: To assess the efficacy of thoracoscopy and the outcome for children with thoracic neurogenic tumors. Methods: We performed a retrospective review of 15 European centers between 2000 and 2020 with patients who underwent thoracoscopy for a neurogenic mediastinal tumor. We assessed preoperative data, complications, and outcomes. Results were expressed with the median and range values. Results: We identified 119 patients with a median age of 4 years old (3 months–17 years). The diameter was 5.7 cm (1.1–15). INRG stage was L1 *n* = 46, L2 *n* = 56, MS *n* = 5, M *n* = 12. Of 69 patients with image-defined risk factors (IDRF), 29 had only (T9–T12) locations. Twenty-three out of 34 patients with preoperative chemotherapy had an 18 mm (7–24) decrease in diameter. Seven out of 31 patients lost their IDRF after chemotherapy. Fourteen had a conversion to thoracotomy. The length of the hospital stay was 4 days (0–46). The main complications included chylothorax (*n* = 7) and pneumothorax (*n* = 5). Long-term complications included Horner’s syndrome (*n* = 5), back pain, and scoliosis (*n* = 5). Pathology was 53 neuroblastomas, 36 ganglioneuromas, and 30 ganglioneuroblastomas. Fourteen had a postoperative residue. With a median follow-up of 21 months (4–195), 9 patients had a recurrence, and 5 died of disease. Relapses were associated with tumor biology, histology, and the need for chemotherapy (*p* = 0.034, <0.001, and 0.015, respectively). Residues were associated with preoperative IDRF (excluding T9–T12 only) and the need for preoperative chemotherapy (*p* = 0.04 and 0.020). Conclusion: Our results show that thoracoscopy is safe, with good outcomes for thoracic neurogenic tumors in selected cases. Surgical outcomes are related to the IDRFs, whereas oncologic outcomes are related to tumor histology and biology.

## 1. Introduction

Neurogenic tumors (NT) comprise neuroblastomas (NBL), ganglioneuroblastomas (GNBL), and ganglioneuromas (GN), and they are the most common extracranial solid tumors in children. They are located on the sympathetic chains that run along the paravertebral wall from the neck to the pelvis. The thoracic location occurs in around 25% of cases and carries a better prognosis owing to its more frequent favorable biological status, with fewer metastatic tumors than abdominal sites [1].

Surgery remains the main treatment for such tumors, as GNs and GNBLs are poorly sensitive to chemotherapy, and resected NBLs have a better prognosis than non-resected NBLs [2,3]. These resections can be performed by either thoracotomy or thoracoscopy. Thoracotomy has been used by the pediatric surgical community for many years and offers the advantages of straightforward access to the tumor and fewer technical skill requirements than thoracoscopy. However, postoperative thoracotomy has the potential for long-term complications, including scoliosis, shoulder elevation, winged scapula, and asymmetric nipples [4]. By comparison, thoracoscopy offers the advantages of less operative blood loss, shorter duration of stay, fewer musculoskeletal complications, and better cosmetic effects than thoracotomy [5,6,7].

Based on these advantages, over the past twenty years, mediastinal NTs have been increasingly resected in line with a minimally invasive approach. Thoracoscopy’s use for neurogenic mediastinal tumors has increased since the first case series in the late 1990s and early 2000s. However, limited data and numbers regarding outcomes, indications, conversion rate, and complications are available. Recently, a SIOPEN study attempted to define guidelines for MIS in NT without detailing the results of 78 patients operated on by thoracoscopy. The study advocated caution in using a thoracoscopic approach for patients with IDRF because the approach presented more complications and incomplete resections than abdominal NT operated on by celioscopy [8,9,10,11]. Other multicenter studies have attempted to better define the place of thoracoscopy on 20 patients according to the presence or absence of image-defined risk factors (IDRF), with a risk of conversion when the tumor is in contact with either the big vessels or trachea [12]. We believe that a study with large numbers would better define the indications of thoracoscopy for mediastinal NT. We aimed to conduct a multicenter retrospective study to assess the role of thoracoscopy in NT according to the presence of IDRF at diagnosis, biology, and pathology of the NT and its impact on the residual disease, risk of conversion, surgical complications, and oncologic outcome.

## 2. Materials and Methods

This retrospective observational anonymized study did not need ethical approval and informed consent (MR-004 according to French bioethics law). A non-opposition form was sent to the patients. The Nice Côte d’Azur University’s IRB approval was F 20210415172320 (www.health-data-hub.fr, access date 15 April 2021), and the clinical trial number was NCT04554173 (www.clinicaltrial.gouv, access date 27 April 2022).

We performed an open survey among the Société Française de Chirurgie Pédiatrique (SFCP), the Groupe de Chirurgiens Opérant des Tumeurs (GCPOT), and the Groupe d’Etude en Célioscopie Infantile (GECI) to spot the centers that had cases of patients under 18 years of age with thoracic neurogenic tumors (NBLs, GNBLs, and GN on final pathology) operated on (i.e., with resection) by thoracoscopy starting from January 2002 to February 2020. Thirteen French and two foreign collaborating centers agreed to enroll their patients in the study. We excluded patients older than 18, patients with peripheral nerve sheath tumors, and patients with only thoracoscopic surgical biopsies. Pediatric surgeons experienced in thoracic and oncological surgery performed the procedures.

We assessed demographic data: the age, location, and size of the lesion at diagnosis and at the time of surgery, the pathology, molecular biology (nMyc and segmental or numeric anomalies) when available, the need for preoperative chemotherapy and its effects on tumor volume, the presence of IDRF at diagnosis and after chemotherapy, the presence of intraspinal extension, and the INRG stage. We assessed operative data: insufflation, the number and size of ports, the need and reasons for conversion to thoracotomy, the intraoperative complications, and the need to divide the lesion. Assessment of postoperative data included immediate and late complications, a residue of more than 5 mL, recurrence, or relapse.

The need for conversion to thoracotomy, the occurrence of operative and postoperative complications, the presence of a postoperative residue, and relapse or recurrence of the disease were analyzed according to the tumor characteristics (preoperative size and chemotherapy, IDRF, pathology, nMyc amplification, array CGH).

Surgical resection by thoracoscopy was performed with the child generally placed in lateral decubitus under general anesthesia with selective bronchial contralateral ventilation using a double-lumen endotracheal tube. Younger children underwent mainstem bronchus intubation with an uncuffed tube or a bronchial blocker. In small children, when it was impossible to perform selective bronchial intubation, the ipsilateral lung was collapsed by inserting low-pressure (around 4 mmHg) carbon dioxide. Three or more trocars were inserted in triangulation. Trocars ranged from 3 to 10 mm, depending on the patient’s size. Zero or 30° optical lenses were used. To perform dissection, two or more other trocars were inserted between the anterior and posterior axillary lines. The pleural tissue covering the mass was incised around the overall circumference of the lesion, and the tumor was released by dissection using either coagulation or thermofusion. The intercostal and vertebral vessels involved with the tumor were occluded with clips, electrocautery, or other energy devices. Once fully released, the tumor was placed in a plastic bag and removed through an enlarged trocar site. In some children, when the tumor was too big, it was morcellated in a plastic bag before removal. In some children, a single chest tube was placed into the thoracic cavity after the procedure, which was aspirated through a four-chamber device. Criteria for removal were the absence of air leak and pleural effusion. In some centers, there was no chest drainage after the procedure.

### Statistics

Results were expressed as median and range values. Comparisons were performed with non-parametric tests. When *p* < 0.1, we used multiple logistic regression to test the variables for the following outcomes: residue, complications, conversion, and recurrence.

Survival analyses were performed for recurrences using the Kaplan–Meier method and Cox proportional regression for multivariate analyses.

*p* < 0.05 was regarded as significant.

## 3. Results

One hundred and nineteen patients were identified in 15 centers, with a median of 8 (1–27) patients per center. Characteristics of the study population and tumors are displayed in Table 1.

Thirty-four (28%) patients had preoperative chemotherapy (Table 2); among the 19 L2 patients with preoperative chemotherapy, 3 (15%) had only the T9–T12 location as an IDRF (1 GN and 2 NBL). Three out of eight patients with either GNBL or GN had a significant decrease (>33%) in their diameter, and three were cleared of their IDRFs. Four (17%) out of the 23 patients with an NB and an IDRF were cleared of preoperative IDRF after chemotherapy. Of the 40 patients with IDRFs with or without preoperative chemotherapy and excluding patients with T9–T12 location only, 30 kept a preoperative IDRF (one patient’s status is unknown).

### 3.1. Surgical Procedures

The median number of trocars used was 3 (3–5). Seventy-six (64%) of the operations were performed with intraoperative insufflation, and 34 (29%) required tumor fragmentation for exteriorization.

The median operative time was 120 (30–345) minutes. Postoperative thoracic drainage was used in 89 (74%) patients with a median duration of 3 (1–46) days. The median hospital length of stay was 4 days (range: 2–46).

### 3.2. Complications

Fourteen (11.7%) patients required conversion to thoracotomy (Table 3). None had any conversion related to the T9–T12 location. Preoperative chemotherapy was significantly associated with conversion to thoracotomy (*p* = 0.039). On the other hand, neither preoperative IDRF (excluding T9–T12) nor preoperative tumor size was associated with conversion (Table 4).

There were no postoperative deaths. Twenty (16.8%) patients presented a postoperative complication (Table 4), of which four were unrelated to surgery: there were two febrile patients without infection, one patient with a urinary tract infection, and one with hypoventilation related to spinal analgesia. There were no neurologic issues in the patients operated on with a T9–T12 location. Long-term complications (after 1 month) occurred in 13 patients (11%) (Table 5). Horner’s and Harlequin syndrome occurred in apical tumors. Only one already had postoperative Horner’s syndrome. Both early and late surgical complications accounted for 25 (21%) patients and were associated with preoperative IDRFs excluding T9–T12 only (Table 4). Two T9–T12-only IDRF patients developed a late complication: scoliosis and chylothorax.

Fourteen (11.7%) had a residue of more than 5 mL (7 NBLs, 5 GNBLs, and 2 GNs). Among these, eight had preoperative chemotherapy, eight had preoperative IDRF excluding T9–T12 only, six were dumbbell tumors, four had vascular IDRF, and one had a bronchus compression. The presence of a preoperative IDRF and the need for preoperative chemotherapy were significantly associated with a postoperative residue (Table 4). The preoperative size was also statistically associated with the presence of a postoperative residue, with an average preoperative size of 70 mm (22–100) in those with a residue vs. 48 mm (11–123) in those without a residue (*p* = 0.001). Six out of 28 dumbbell tumors had a residue vs. 8 out of 91 patients without (*p* = 0.074). Among the patients who had surgery despite remaining vascular and/or bronchial IDRF, there was a significantly increased incidence of postoperative residue compared with the 101 without bronchial vascular IDRF (Table 6).

**Figure 1:**
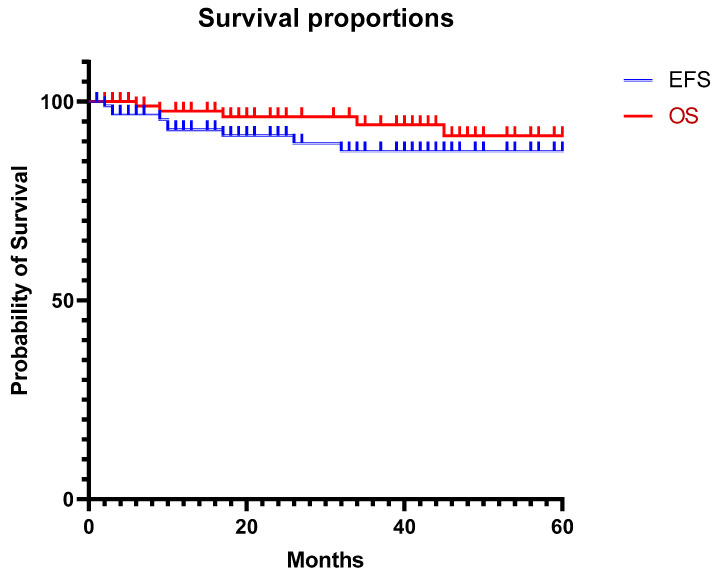
Survival curves. EFS: event-free survival, OS: overall survival.

With a median follow-up of 21 months (4–195), nine (7.5%) patients presented with a relapse (five local and metastatic, two local, two metastatic) at a median of 10 months (2–32). All presented with NBLs. INRG stage was 4 M, 3L1, and 2L2. Among the three L1 patients who relapsed locally, although two had synchronous metastases, two had segmental anomalies (1 year and 5 years old at diagnosis), and for another one, the CGH was not performed (operated on in 2011). The L1 patient with the localized relapse had a fragmentation during the thoracoscopy, requiring conversion to thoracotomy; the tumor harbored a CHG segmental anomaly. For the L2 patients, one had fragmentation during the thoracoscopy, and the tumor was nMyc amplified; he had a localized and metastatic relapse. The other, 3 years old at surgery, had a segmental anomaly and had a local relapse. Only one (metastatic) patient with relapse had a postoperative residue after thoracoscopy. Five patients died of their disease 7 (0–13) months after their relapses (Figure 1). Both NBL histology and the need for preoperative chemotherapy were statistically associated with relapse (*p* = 0.017 and *p* = 0.015). Separately, neither segmental profile nor nMyc amplification was associated with relapses (respectively *p* = 0.061 and *p* = 0.106) (Table 1). However, segmental profile or nMyc amplification put together and regarded as unfavorable tumor biology was associated with relapse (*p* = 0.034). The postoperative residue was unrelated to relapse (*p* = 0.487) (Table 3). Eighteen patients had vascular or bronchial IDRFs. These challenging IDRFs were associated only with residue (*p* = 0.032) but not complication, conversion, or relapse (Table 5). There were no more complications (5 vs. 20) or relapses (2 vs. 7) in patients who had a conversion to thoracotomy vs. those who had not (*p* = 0.091 and 0.178, respectively).

On multivariate analysis, neither the preoperative IDRFS (excluding T9–T12) nor prior chemotherapy nor dumbbell tumor was associated with the presence of a residue (*p* = 0.053, *p* = 0.120, and *p* = 0.788, respectively). The results were the same with the conversion rate (*p* = 0.375 and 0.159). Regarding relapses, only unfavorable biology (nMyc or segmental anomalies) was significantly associated with relapses; odds ratio: 22.0 95% CI: 1.8–99.1, and *p* = 0.0376. Neuroblastoma histology could not be tested because all relapses appeared in this category. With survival analyses, Cox proportional hazard regression confirmed these results (HR: 9.776, 95% CI: 1.320–214).

## 4. Discussion

Thoracoscopic resection of neurogenic tumors is a rare and challenging situation in pediatric surgical oncology, as the median of patients per center by year was less than 0.5 and 1.5 for the highest volume hospital in this series. This is why multicenter studies are necessary to refine the indications.

No studies so far have grouped more than 100 patients in 15 centers operated on for neurogenic mediastinal or thoracic tumor resection to allow robust analysis of conversions, complications, and outcomes (Table 7).

Regarding the technique, the 2 h operative time, the 75% rate of chest drainage, and the length of hospital stay were comparable to the existing literature on thoracoscopy [8,9,10,13,14,15,16]. This can be an argument to encourage use in pediatric surgical oncology.

Despite large tumors at operation (median: 57.1 mm), our 11.7% conversion rate was low. In the literature, the conversion rate ranged from 0% to 14% and could have been under-reported in small series. Our series found an association between conversion and neoadjuvant chemotherapy, although the conversion rates reported so far were associated with IDRF [8,13,14,15,17]. This association disappeared in multivariate analysis. The decision to give neoadjuvant chemotherapy is based on histology (NBL vs. GN/GNBL) and IDRFs. Our study’s population was comparable to the literature that had already been published, with 43–59% of NBL patients among thoracic neurogenic tumors [8,10]. When they received preoperative chemotherapy, the diameter decreased significantly, which may have allowed a high resection rate by thoracoscopy. However, if we exclude “T9–T12 only” IDRF, 22% of the patients cleared their IDRF after chemotherapy. Moreover, chemotherapy before surgery was not associated with a lower rate of complications but was associated with a higher rate of residues, conversion, and relapses. This result had not been reported previously [8,9,10,11,12,13,14,15,17]. We assume that the need for preoperative chemotherapy is a hallmark of aggressive tumor behavior, as tumor histology and biology somewhat influenced the outcome of our multivariate analysis. These results should advocate caution in using the minimally invasive approach for patients needing chemotherapy before surgery. We do not support the use of chemotherapy outside the scope of the protocol to avoid conversion and complications of the thoracoscopic approach. However, more surprisingly, a few patients with GNBLs may have benefited from chemotherapy to clear their IDRFs. This contradicts other studies pointing out the non-efficacy of chemotherapy on GN/GNBLs [3]. Of note, the location in the inferior mediastinum between T9–T12 is regarded as an IDRF (because of the Adamkiewicz artery location) but was not a limitation for the thoracoscopic approach in our study. No patient with T9–T12 location as the only IDRF required conversion to thoracotomy or had postoperative complications, although two patients had late complications unrelated to the Adamkiewicz artery.

Our postoperative and long-term complication rates (16.8% and 11%, respectively) were comparable to the 17–30% of other existing series [8,9,10,11,12,13,14,15,17]. Horner’s syndrome, chylothorax, back pain, and long-term scoliosis are the main complications of this approach but have a similar rate to open thoracic approaches [8,9,10,11,12,13,14,15,17]. These complications are somewhat related to the origin of the tumor: dumbbell tumors requiring neurosurgical procedures or laminectomies for back issues and apex location (stellate ganglia) for Horner’s or Harlequin syndrome. In our study, the IDRF was the only factor influencing this complication rate.

Complete resection (i.e., residue less than 5 mL) was achieved in 88% of cases, consistent with scarce reports about the residue in MIS for neurogenic tumors [8]. In our study, the presence of postoperative residue was associated with preoperative chemotherapy and bronchial and vascular IDRFs, both factors that may have been linked. Another risk factor for postoperative residue was tumor size, which was not published in this localization before. However, recently, an American Pediatric Surgical Association recommendation based only on a survey and a review showed that a minimally invasive approach is suitable for tumors without IDRF and with sizes of 4–6 cm, therefore supporting our findings [17]. Other studies showed that the residue is related to the INRG staging and IDRFs, supporting our results [8,16]. Our study focused on preoperative IDRF, apart from the T9–T12 only location, given the exceptional complications related to the Adamkiewicz artery and because the T9–T12 location should persist despite chemotherapy [18]. This approach allowed us better to analyze surgical difficulties and their impact on outcomes. The clearance of IDRFs was 22%, less than the 43% reported in a recent comparative study on thoracotomy and thoracoscopy for NBL [16]. An interesting finding from our study is that residues were not associated with relapses. Other studies on pelvic location showed the same results regarding residue and outcome. These tumor locations have in common a typically good biology [19,20]. Our study showed a low rate of relapses, which was expected regarding the good prognosis of this localization, and the high rate of GN and GNBL (one relapse in this histology). All the other relapses occurred in NBs, which needed chemotherapy.

Similarly, the biology (nMyc or segmental profile) appeared to have more impact on relapses than residue itself. Moreover, the relapses in L1 patients with previous tumor fragmentation should encourage avoiding fragmentation or non-protected extraction during primary surgery in case of possible non-favorable biology, which occurred in 12 of the patients in our study. Although the missing data in our study necessitate caution in interpreting these results, large studies on CGH segmental anomalies showed a higher risk of relapses [21].

With the implementation of thoracic robotic surgery with 3D imaging technology, which provides high-quality magnified images and 3D complex movements in enclosed spaces, surgeons may be able to approach mediastinal neurogenic tumors with bronchial or vascular attachment or more complex IDRFs in the future. To our knowledge, only preliminary results have been published in the adult population so far [22]. Conversely, small neurogenic tumors without IDRFs could be approached using uniportal video-assisted thoracic surgery, although pediatric surgeons have not adopted this technique on a large scale [23].

The main limitation is that our study is retrospective and not comparative. On the basis of our study, it is impossible to conclude that thoracoscopy has advantages over thoracotomy. Only a study comparing thoracoscopy and thoracotomy groups could enable such conclusions. In small retrospective studies, the benefits of thoracoscopy were only demonstrated on the length of drainage and length of stay [10,11]. Given the small number of patients operated on for this type of cancer, a prospective study is practically impossible. However, it would be possible to perform a retrospective study with some attempt to control bias, for example by using propensity score matching studies.

## 5. Conclusions

This large series demonstrated that thoracoscopic resections for neurogenic tumors had a rate of 11.7% regarding conversion, long-term complication, and residue and a relapse rate of 7.5%. The risk for conversion is associated with the need for chemotherapy, complications are related to IDRFs, and residues are associated with persisting bronchial or vascular IDRF. Recurrences are associated with neuroblastoma histology and unfavorable tumor biology. We therefore recommend caution in using thoracoscopy for patients needing preoperative chemotherapy (apart from MS patients) and IDRFs (apart from T9–T12). Future studies focusing on these two issues could be conducted.

## Figures and Tables

**Table 1:** Characteristics of the study population and tumors (IDRF: image-defined risk factor, CGH: array comparative genomic hybridization, INRG: international neuroblastoma risk group stratification).

Characteristics of the Study Population	Median/*n*	(Extremes) or (%)
Age	4 years	(3 months–17 years)
Left location	58	(49%)
INRG staging		
L1	46	
L2	56	
M	12	
MS	5	
IDRF	69	(58%)
T9–T12 location as only IDRF	29	
Extension to the medullary canal	28	
Encasement of aorta/subclavian artery	14	
Compression of trachea/bronchus	6	
Diameter		
At diagnosis	57.1 mm	(11–150)
At surgery	51.6 mm	(11–123)
Histopathology		
Neuroblastoma	53	(45%)
Ganglioneuroblastoma	30	(30%)
Ganglioneuroma	36	(30%)
Tumor biology		
nMyc amplification	2	
Segmental alteration on CGH	11	
Numeric alteration on CGH	13	

**Table 2:** Patients with preoperative chemotherapy.

Patients with preoperative chemotherapy *n* = 34		
INRG staging		
L1	0	
L2	19	
MS	3	
M	12	
Pathology		
NBL	26	
GNBL	6	
GN	2	
	Pre CT	Post CT
Size	71 mm (30–50)	55 mm (11–120)
IDRF	31	24

INRG: international neuroblastoma risk group stratification; NBL: neuroblastoma; GNBL: ganglioneuroblastoma; GN: ganglioneuroma; IDRF: image-defined risk factors. CT: chemotherapy

**Table 3:** Reasons for conversions.

Conversions	*n* = 14
Vascular attachment	6
Lack of space	5
Friable tumor	2
Need for pulm. resection	1

**Table 4:** Postoperative results depending on tumor characteristics.

			Residue		Complication		Conversion		Relapse	
			+ 14 (11.7%)	− 105 (88.2%)	+ 25 (21%)	− 94 (79%)	+ 14 (11.7%)	− 105 (88.2%)	+ 9 (7.5%)	− 110 (92.4%)
CGH/nMyc	Fav	13	3	9	3	10	2	11	**1**	**12**
	Unfav	12	3	10	2	10	2	10	**5**	**7**
	N/A	94		*p* = 0.99		*p* = 0.724		*p* = 0.930		***p* = 0.034**
Histology	NB	53	7	46	9	44	6	47	**9**	**44**
	GN/B	66	7	59	16	50	8	58	**0**	**66**
				*p* = 0.33		*p* = 0.173		*p* = 0.903		***p* < 0.001**
IDRF *	+	30	**8**	**22**	**10**	**20**	6	24	4	26
	− N/A	88 1	**6**	**82** ***p* = 0.004**	**15**	**73** ***p* = 0.036**	8	80 *p* = 0.063	5	83 *p* = 0.105
Pre-op ChemoT	+	34	**8**	**26**	8	26	**7**	**27**	**6**	**28**
	−	85	**6**	**79 ** ***p* = 0.020**	17	68 *p* = 0.33	**7**	**78** ***p* = 0.039**	**3**	**82 ** ***p* = 0.015**

CGH: array comparative genomic hybridization; Fav: both numeric and nMyc not amplified; Unfav: either segmental profile or nMyc amplified; NB: neuroblastoma; N/A: not performed; GN/B: ganglioneuromas and ganglioneuroblastomas; IDRF *: preoperative image-defined risk factor excluding T9–T12 location; Pre-op ChemoT: preoperative chemotherapy; Bold: it is statitistically significant on monovariate analysis.

**Table 5:** Complications (some patients experienced several complications).

	Post Operative *n* = 20	Long Term *n* = 13
Medical	4	
Pneumothorax	4	
Requiring new drainage	3	
Horner’s syndrome	4	6
Chylothorax	7	1
Requiring surgery	3	0
Harlequin syndrome		1
Scoliosis		4
Chronic back pain		3

**Table 6:** Vascular and/or bronchial IDRF analysis.

		Vascular or Bronchial IDRF		*p*
		+ 18 (15.1%)	− 101 (84.8%)	
Complication	+15 (12.6%) −104 (87.3%)	2 16	13 88	*p* = 0.445
Conversion	+14 (11.7%) −105 (88.2%)	4 14	10 91	*p* = 0.087
Residue	+14 (11.7%) −105 (88.2%)	**5** **13**	**9** **92**	**(*p* = 0.037)**
Relapse	+9 (7.5%) −110 (92.4%)	2 16	7 94	*p* = 0.272

Bold: it is significant.

**Table 7:** Review of the literature on thoracoscopic approach for neurogenic mediastinal tumors.

Authors	Type of Study	N	Conversion	Complications	Residue	Recurrence	Remarks
Lacreuse et al. 2007 [9]	Multicenter	21	1 (4%)	6 (28%)	0	0	Few comments on the outcomes
Gabra et al. [8]	Multicenter	78	11 (14%)	NR	33 (42%)	NR	
Malek et al. [10]	Monocentric comparative	11	NR	3 (11%)	NR	1 (9%)	
Fraga et al. [13]	Multicenter	17	0	2 (11%)	NR	0	
Petty et al. [14]	Monocentric comparative	10	0	2 (20%)	NR	1 (10%)	
Kawano et al. [15]	Multicenter	28	0	1 (5%)	NR	NR	Few outcomes reports
Delforge [16]	Multicenter comparative	9	NR	NR	NR	NR	Few statistics
Present study	Multicenter	114	14 (11.7%)	25 (21%)	14 (11.7%)	9 (7.5%)	

NR: not reported.

## Data Availability

Data are available on request to the authors.
